# A shared mechanism for TNP-ATP recognition by members of the P2X receptor family

**DOI:** 10.1016/j.csbj.2023.12.005

**Published:** 2023-12-07

**Authors:** Xiao-Bo Ma, Chen-Xi Yue, Yan Liu, Yang Yang, Jin Wang, Xiao-Na Yang, Li-Dong Huang, Michael X. Zhu, Motoyuki Hattori, Chang-Zhu Li, Ye Yu, Chang-Run Guo

**Affiliations:** a Department of Pharmacology and Chemical Biology, Institute of Medical Sciences, Shanghai Jiao Tong University School of Medicine, Shanghai 200025, China; b School of Basic Medicine and Clinical Pharmacy, and State Key Laboratory of Natural Medicines, China Pharmaceutical University, Nanjing 210009, China; c State Key Laboratory of Utilization of Woody Oil Resource, Hunan Academy of Forestry, Changsha 410004, China; d Department of Integrative Biology and Pharmacology, McGovern Medical School, The University of Texas Health Science Center at Houston, Houston, TX 77030, USA; e State Key Laboratory of Genetic Engineering, Collaborative Innovation Center of Genetics and Development, Shanghai Key Laboratory of Bioactive Small Molecules, Department of Physiology and Neurobiology, School of Life Sciences, Fudan University, Shanghai 200438, China; f School of Traditional Chinese Pharmacy, and State Key Laboratory of Natural Medicines, China Pharmaceutical University, Nanjing 210009, China

**Keywords:** TNP-ATP, P2X3 receptor, P2X7 receptor, Recognition mechanism

## Abstract

P2X receptors (P2X1–7) are non-selective cation channels involved in many physiological activities such as synaptic transmission, immunological modulation, and cardiovascular function. These receptors share a conserved mechanism to sense extracellular ATP. TNP-ATP is an ATP derivative acting as a nonselective competitive P2X antagonist. Understanding how it occupies the orthosteric site in the absence of agonism may help reveal the key allostery during P2X gating. However, TNP-ATP/P2X complexes (TNP-ATP/human P2X3 (hP2X3) and TNP-ATP/chicken P2X7 (ckP2X7)) with distinct conformations and different mechanisms of action have been proposed. Whether these represent species and subtype variations or experimental differences remains unclear. Here, we show that a common mechanism of TNP-ATP recognition exists for the P2X family members by combining enhanced conformation sampling, engineered disulfide bond analysis, and covalent occupancy. In this model, the polar triphosphate moiety of TNP-ATP interacts with the orthosteric site, while its TNP-moiety is deeply embedded in the head and dorsal fin (DF) interface, creating a restrictive allostery in these two domains that results in a partly enlarged yet ion-impermeable pore. Similar results were obtained from multiple P2X subtypes of different species, including ckP2X7, hP2X3, rat P2X2 (rP2X2), and human P2X1 (hP2X1). Thus, TNP-ATP uses a common mechanism for P2X recognition and modulation by restricting the movements of the head and DF domains which are essential for P2X activation. This knowledge is applicable to the development of new P2X inhibitors.

## Graphical Abstract

**Figure:**
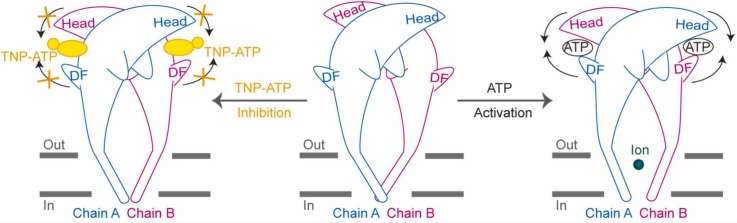

## 1 Introduction

P2X receptors are non-selective cationic ion channels activated by extracellular ATP [1], [2], [3]. A total of seven subtypes, including P2X1–7, have been cloned. In mammals, they are present as homo- or hetero- trimers on both the plasma membrane and intracellular organelles, for example lysosomes [4], [5], [6]. P2X receptors are expressed in most tissues and are involved in a variety of physiological and pathological processes, including synaptic transmission, smooth muscle contraction, platelet aggregation, injury perception, touch, taste, hearing, hypertension, immune regulation, and tumor proliferation [7], [8], [9], [10], [11], [12], [13], [14], [15], [16], [17]. A few antagonists targeting the P2X receptors have been marketed or have passed through major clinical trials and shown positive therapeutic effects. For instance, P2X7 antagonists, CE-224,535 and AZD90564 (rheumatoid arthritis), GSK-1482160 (inflammation), AFC-5128 (neuropathic pain, multiple sclerosis and gastrocnemius dystrophy), JNJ-54175446 (major depressive disorder), JNJ-55308942 (mood disorder), SGM-1019 (nonalcoholic steatohepatitis), BIL010t (melanoma) and RQ-00466479 (neuropathic pain), are in Phase I or Phase II clinical trials; a P2X4 antagonist, NC-2600, is in clinical trials for neuropathic pain; and a P2X3 inhibitor, AF-219, is now marketed in Japan for the treatment of refractory chronic cough. Thus, there are great potentials for drug development targeting P2X receptors [18], [19], [20], [21], [22]. 2',3'-O-(2,4,6-trinitrophenyl)-ATP (TNP-ATP) belongs to a class of non-selective competitive inhibitors of the P2X receptors. As a derivative of ATP, TNP-ATP was initially used as a fluorescent marker of ATP [23]. It was subsequently identified as a potent antagonist of all P2X receptors, particularly homomeric P2X1, homomeric P2X3, and heterotrimeric P2X2/3 [24], [25], [26]. Later, TNP-ATP and other 2',3'-O-substituted ATP derivatives were classified as potent P2X inhibitors [27].

ATP-induced activation has been studied intensively by means of structural and functional biology for P2X receptors of various species and subtypes [28], [29], [30], [31], [32], [33], [34]. These studies reveal very conserved ATP recognition and activation mechanisms for the P2X receptor family members (ATP act mainly in the cavity formed by two adjacent subunits, and interacted with the head, dorsal fin (DF) and left flipper (LF) domains), *e.g.*, the close similarity between Gulf coast tick P2X receptor (*Amblyomma maculatum* P2X, AmP2X) [31] and human P2X3 (hP2X3) [28], not to mention the highly conserved ATP recognition and gating mechanism among mammalian panda P2X7 (pdP2X7) [30], rat P2X7 (rP2X7) [32] and hP2X3 [28]. However, some of the allosteric mechanisms are not so obvious when comparing the open and closed structures, such as the upper body domain and the upper vestibule, which are rigid architectures [30], [35]; completely different binding conformations were obtained for different P2X receptors bound by the same inhibitor, *e.g.*, TNP-ATP [28], [36].

Currently, two TNP-ATP-bound structures of P2X receptors have been determined, namely hP2X3 (PDB ID: 5SVQ) and chicken P2X7 (ckP2X7, PDB ID: 5XW6) [28], [36]. In contrast to the highly conserved mechanism of P2X receptor activation by ATP, these two structures show completely different overall conformations and TNP-ATP recognition mechanisms. The TNP-ATP-bound ckP2X7 exhibits an incompletely activated pore conformation (the transmembrane (TM) region is extended outward, but the channel pore is still impermeable to ions). Moreover, its extracellular domain shows an extended shape somewhat similar to the open state of the P2X receptor [28], [29], [31], [36], which resembles the TNP-ATP-bound zebra fish P2X4 (zfP2X4) by NMR analysis [37]. In addition, TNP-ATP in this structure exhibits a "U-shaped" occupancy pose resembling ATP binding [28], [29], [30], [31], with its 2,4,6 trinitrophenyl (TNP) moiety interacting with amino acid residues in the head and DF domains of the receptor. This binding mode is somewhat consistent with a previous result on the P2X1 receptor by voltage clamp fluorometry analysis [34]. In contrast, the structure of the hP2X3 receptor bound by TNP-ATP resembles the *apo* or closed conformation of P2X [28], [38]. In this structure, the TNP-ATP molecule shows a "Y-shaped" occupancy and its TNP moiety is rotated ∼180° toward the lower body (LB) domain of P2X3 and is encapsulated by the LF region [28]. These two different TNP-ATP binding modes might arise from subtype or species differences, which would be inconsistent with the highly conserved ATP-binding mode across different species and subtypes of P2X receptors [28], [29], [30], [31], [32], [33], [34]. Alternatively, the final binding conformation of TNP-ATP to the P2X family members may be very similar, and either or both of determined structures could represent intermediate states. Moreover, it cannot be ruled out that the differences might simply arise from the different experimental approaches used.

Here, we evaluated the interaction of TNP-ATP with P2X receptors based on the above two structures by combining enhanced conformational sampling, covalent occupation, and engineered disulfide bond analysis. Our results reveal a general mechanism for the interaction of TNP-ATP with the P2X receptors, in which the polar triphosphate group of TNP-ATP interacts with the orthosteric site, while its TNP moiety is deeply embedded in the interface between the head and DF domains, which restricts the conformational changes associated with channel opening in these two domains. In addition, the TM region, which is indirectly coupled to the DF domain *via* the body domain, has an outward motion but no ion permeation in the final state. Our model generally agrees with the binding mode revealed by the ckP2X7/TNP-ATP structure complex, but has an important refinement, in which V130 and H131 of ckP2X7, instead of T202 and T112 as revealed by structural biology, are key sites for TNP-ATP recognition. We confirmed this binding mode in hP2X3, rP2X2 and hP2X1, demonstrating that it represents a common recognition mechanism of the P2X receptors to this class of non-selective competitive inhibitors, which should be instructive to future designs of P2X receptor drugs.

## 2 Materials and methods

### 2.1 Chemicals and mutagenesis

Unless otherwise stated, all compounds were purchased from Sigma-Aldrich (USA). TNP-ATP triethylammonium salt was purchased from Tocris bioscience (Catalog No.: 2464), with a purity of more than 98%. hP2X3 plasmid was purchased from Open Biosystems; ckP2X7 cDNA was a generous gift of Dr. Osamu Nureki and was subcloned into pcDNA3.1 vector; pcDNA3-rP2X2, pcDNA3-rP2X3 plasmids were generous gifts of Drs. Alan North and Linhua Jiang. All mutants were created using the KOD-Plus- mutagenesis kit (Toyobo, SMK-101) and confirmed by DNA sequencing [39].

### 2.2 Cell culture and electrophysiology

As we described previously [40], [41], [42], human embryonic kidney (HEK 293) cells were purchased from Shanghai Institutes for Biological Sciences and cultured in Dulbecco's Modified Eagle Medium supplemented with 10% fetal bovine serum (FBS), 1% penicillin-streptomycin and 1% GlutaMAX™ at 37 °C, 5% CO_2_ and 95% air in a humidified environment. Plasmids were transfected into cells using transfection agents containing two solutions (Solution A: 250 mM CaCl_2_ in pure water; Solution B (in mM): 1.5 Na_2_HPO_4_, 140 NaCl and 50 HEPES, pH adjusted to 6.96). After mixing 2.5–3 μg of the plasmid and solution A, the mixture was added dropwise to an equal volume of solution B, followed by stirring with a pipette tip. The mixture was left to stabilize at room temperature for 3–5 min and then added to the cell culture dish. After 6 h of transfection, the medium in the culture dish was replaced with fresh medium.

As described previously [43], ionic currents of hP2X3 and rP2X3 were recorded using Nystatin (Sangon Biotech) perforated patches to avoid current rundown during multi-dose application of ATP. Nystatin (0.15 mg/mL) was diluted with a high potassium internal solution containing (in mM): 75 K_2_SO_4_, 55 KCl, 5 MgSO_4_, and 10 HEPES (pH 7.4). The ckP2X7 and rP2X2 receptor currents were recorded using conventional whole-cell patch configuration. At 24–48 h after transfection, HEK293 cells were recorded using an Axopatch 200B amplifier (Molecular Devices, USA) at room temperature (25 ± 2 °C), with holding a potential of − 60 mV. Current data were sampled at 10 kHz, filtered at 2 kHz, and analyzed using the PCLAMP 10 software (Molecular Devices, USA). HEK293 cells were bathed in standard extracellular solution (SS) containing (in mM): 150 NaCl, 5 KCl, 2 CaCl_2_, 1 MgCl_2_, 10 glucose, and 10 HEPES (pH 7.4, by Tris-base). For conventional whole-cell recordings, the pipette solution consisted of (in mM): 120 KCl, 30 NaCl, 0.5 CaCl_2_, 1 MgCl_2_, 5 EGTA, and 10 HEPES (pH 7.4, by Tris-base). ATP and drugs were dissolved in standard extracellular solution and administered with a Y-tube.

### 2.3 Engineered disulfide bond cross-linking and gel analysis

HEK293 cells were transfected with rP2X3-WT or mutant plasmids, washed three times with phosphate buffered saline (PBS; pH 7.4), and then incubated with 2 mL sulfo-NHS-LC-biotin (Pierce, Germany). The cells were then placed in a refrigerator at 4 °C for 30 min with shaking and agitation every 10 min to label membrane proteins on the cell surface. Subsequently, glycine was added to terminate the reaction. Cells were washed three times with PBS, and then RIPA lysis buffer (200 μL) was added. Cell lysates were then collected from the bottom of the culture dish with a cell spatula and then centrifuged at 12,000 rpm for 30 min at 4 °C. Then, 20% (v/v) of the supernatant was added to SDS loading buffer to determine total protein content, with (for reducing gel analysis) and without (for non-reducing gel analysis) β-mercaptoethanol (β-Me), followed by a 5-minute metal bath. Anti-EGFP (1:3000; Sigma, United States) antibodies were incubated overnight at 4 °C. After washing, the blot was incubated with secondary Goat anti-mouse IgG (H+L) HRP (Sungene Biotech, China) for 2 h at room temperature. Finally, the blot was visualized by exposure with automated chemiluminescence-fluorescence image analysis systems (Tanon 5200, Multi) for 1–3 min in the ECL solution (Thermo). Analysis of protein expression was repeated by at least three independent experiments.

### 2.4 Conventional molecular dynamics (CMD) simulations and metadynamics (MetaD) simulations

The energy-minimized structures of hP2X3/TNP-ATP (PDB ID:5SVQ), ckP2X7/TNP-ATP (PDB ID:5XW6) were used as the initial structures for CMD simulations. A large 1-palmitoyl- 2-oleoyl-sn-glycero-3-phosphocholine (POPC, 300 K) bilayer, available in System Builder of DESMOND [42], [44], was built to generate a suitable membrane system based on the OPM database [45]. The hP2X3/TNP-ATP/POPC and ckP2X7/TNP-ATP/POPC systems were dissolved in simple point charge (SPC) water molecules. The DESMOND default relaxation protocol was applied to each system prior to the simulation run. 1) 100 ps simulations in the NVT (constant number of particles (N), volume (V), and temperature (T)) ensemble with Brownian kinetics using a temperature of 10 K with solute heavy atoms constrained; 2) 12 ps simulations in the NVT ensemble using a Berendsen thermostat with a temperature of 10 K and small-time steps with solute heavy atoms constrained; 3) 12 ps simulations in the NPT (constant number of particles (N), pressure (P), and temperature (T)) ensemble using a Berendsen thermostat and barostat for 12 ps simulations at 10 K and 1 atm, with solute heavy atoms constrained; 4) 12 ps simulations in the NPT ensemble using a Berendsen thermostat and barostat at 300 K and 1 atm with solute heavy atoms constrained; 5) 24 ps simulations in the NPT ensemble using a Berendsen thermostat and barostat at 300 K and 1 atm without constraint. After equilibration, MD simulations were performed for ∼0.5–1.0 µs. Long-range electrostatic interactions were calculated using the smooth particle grid Ewald method. Trajectory recording intervals were set to 100–200 ps and for other settings default parameters of DESMOND were used in the CMD simulation runs. All simulations used the all-atom OPLS_2005 force field [46], [47], which is used for proteins, ions, lipids and SPC waters. The Simulation Interaction Diagram (SID) module in DESMOND was used for exploring the interaction analysis between TNP-ATP and hP2X3/ckP2X7.

MetaD simulations were performed by DESMOND under NPT and periodic boundary conditions using the default parameters at constant temperature (330 K) and pressure (1 bar). The parameters for height, width of the Gaussian, and the interval were set to 0.12 kcal/mol, 0.05 Å and 0.09 ps, respectively. The DESMOND default relaxation protocol was applied to each system prior to the MetaD simulation run (same steps as for CMD simulations, see above). All MetaD simulations were lasted for 120 ns until they showed free diffusion along the defined CV. The sum of the Gaussians and the free energy surface (FES) were generated by METADYNAMICS ANALYSIS tools of DESMOMD. The bias V (s, t) is typically constructed in the form of periodically added repulsive Gaussians, where s is the chosen CV which could be multidimensional. Therefore, the free energy surface can be constructed in the space spanned by those CVs. The bias potential *V (s, t)* at time *t* can be written as:

$$V\left(s,t\right)=\int_{0}^{t}\omega \exp\left(-\sum_{i=1}^{d}\frac{{\left(S_{i}-S_{i}\left(t^{\prime }\right)\right)}^{2}}{2\sigma_{i}^{2}}\right)dt^{\prime }$$

where ω is the Gaussian height controlled by the deposition stride, Si is one of d CVs, and σ_i_ is the Gaussian width. This method pushes the system to escape the local minima to find the nearest saddle point on the FES. When the transient happens, the bias provides the free-energy estimate as:

^V(s^, t^)^ = −F^(S)^ + C

where *C* is an arbitrary additive constant, and F(S) is free energy. Since the absolute free energy is normally not important, this constant can be readily eliminated for calculating the free-energy difference. All simulations were performed on DELL T7920 with NVIDIA TESLA K40C or CAOWEI 4028GR with NVIDIA TESLA K80. The simulation system was prepared, trajectory analyzed and visualized on a DELL T7500 graphic workstation with 12 CPUs.

### 2.5 Data analysis

All electrophysiological recordings were analyzed using Clampfit 10.6 (Molecular Devices). Pooled data were expressed as mean and standard error of the mean (s.e.m.). Comparisons between multiple independent groups were performed using one-way ANOVA followed by Dunnett's tests. Comparisons between two groups were made using paired (before and after engineered disulfide bond cross-linking) or unpaired Student’s *t* tests, as appropriate. The dose-response curves were fitted using the Hill 1 equation: I/I_max_ = 1/[1 + (EC_50_/[ATP])^n^], where *I* is the normalized current at a given concentration of ATP, *I_max_* is the maximum normalized current, *EC_50_* is the concentration of ATP yielding one half of maximal currents, and *n* is the Hill coefficient. The inhibitory concentration-effect curves were fitted using the Hill 1 equation: I/I_max_ = 1/[1+(IC_50_/[TNP- ATP])^n^], where *I* is the normalized current at a given antagonist concentration, *I_max_* is the maximum normalized current induced by the agonist, *IC_50_* is the concentration of the antagonist exhibiting the half-maximum effect, *[TNP-ATP]* is the concentration of TNP-ATP, and *n* is the Hill 1 coefficient.

## 3 Results

### 3.1 Comparison of two modes of TNP-ATP recognition by P2X receptors and their stability during conventional molecular dynamics (CMD) simulations

**Fig. 1:**
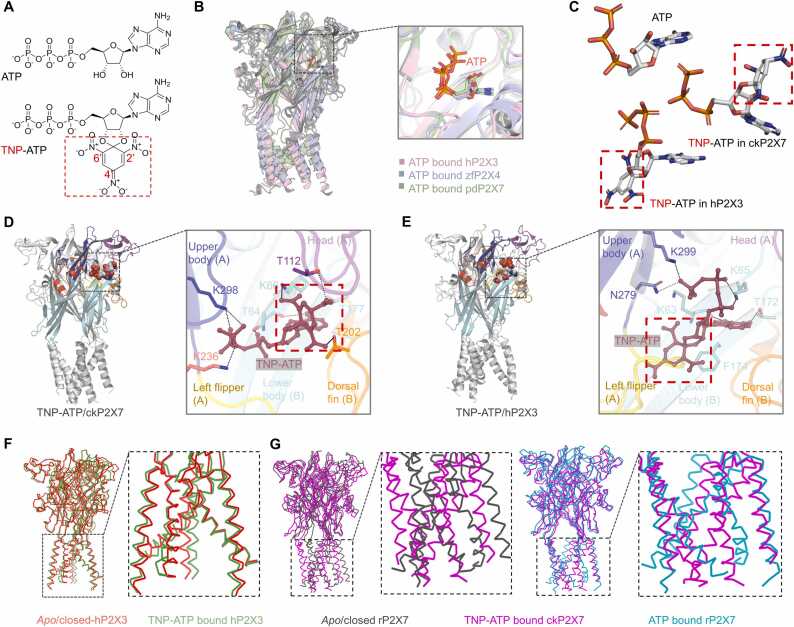
**Comparison of two modes of TNP-ATP recognition by chicken P2X7 (ckP2X7) and human P2X3 (hP2X3)**. (**A**) Chemical structures of ATP and TNP-ATP molecules. (**B**) Binding modes of ATP in hP2X3 (pink), zfP2X4 (indigo) and pdP2X7 (green); PDB IDs: 5SVK, 4DW1, and 5U2H for hP2X3, zfP2X4 and pdP2X7, respectively. (**C**) The orientation of ATP and TNP-ATP in the determined P2X structures. (**D, E**) The bound TNP-ATP conformations in ckP2X7 (D; PDB ID: 5XW6) and hP2X3 (E; PDB ID, 5SVQ). (**F**) Superimposition of TNP-ATP-bound hP2X3 (green) and *apo*/closed hP2X3 (orange). (**G**) Superimposition of TNP-ATP-bound ckP2X7 (magenta) and *apo*/closed (dark, PDB ID: 6U9V) or ATP-bound rP2X7 (cyan, PDB ID: 6U9W).

The P2X receptor structure has a grail-shaped trimeric structure, and each subunit consists of a large hydrophilic extracellular domain, two TM helices, and intracellular termini [28], [29], [30], [31], [32], [37], [38]. ATP and its derivative TNP-ATP (Fig. 1A) act mainly in the cavity formed by two adjacent subunits. Despite the differences in subtypes and species, the ATP molecule adopts almost the same binding mode in the zfP2X4, hP2X3 and pdP2X7 receptors, with an overall "U-shaped" insertion (Fig. 1B, C). For TNP-ATP, two different mechanisms of action have been proposed, with differences in the recognition mode and the overall conformational changes induced by TNP-ATP (Fig. 1C- G).

For recognition, in the ckP2X7/TNP-ATP structure complex (PDB ID: 5XW6), TNP-ATP has a "U-shaped" orientation, like the ATP molecule shown in Fig. 1B, C. The inhibitory TNP moiety interacts with the head, LB and DF domains of the ckP2X7 receptor, forming strong hydrogen bonds (H-bonds) with the amino acids ^ck7^K66, ^ck7^T112 and ^ck7^T202 (the upper corner mark ^ck7^ indicates the sequence number of ckP2X7, hereinafter), and it affects the opening of the channel by preventing the head and DF domains from approaching each other (Fig. 1C, D).

In contrast, in the structure of TNP-ATP-bound hP2X3 (PDB ID: 5SVQ), TNP-ATP exhibits a "Y-shaped" orientation (Fig. 1C, E), with the TNP group rotated approximately 180° toward the LB and LF domains, preventing the downward movement of the LF domain, which is required for channel opening. Interestingly, the TNP group here does not form any strong H- bonds with the hP2X3 receptor, but only interacts with ^h3^F174 in a hydrophobic manner (Fig. 1E), despite the fact that several polar residues in the LF domain are sufficiently close to TNP-ATP.

The changes in the overall structure of the P2X receptor induced by TNP-ATP binding are also inconsistent. The overall structure of the TNP-ATP-bound hP2X3 resembles the *apo*/closed state of hP2X3, which is particularly evident in the TM region (Fig. 1F), *i.e*., no change at all; whereas that of TNP-ATP-bound ckP2X7 exhibits a conformation that is somewhat suggestive of an incomplete open state as it exhibits features of both the *apo*/closed state and ATP-bound open state (Fig. 1G).

**Fig. 2:**
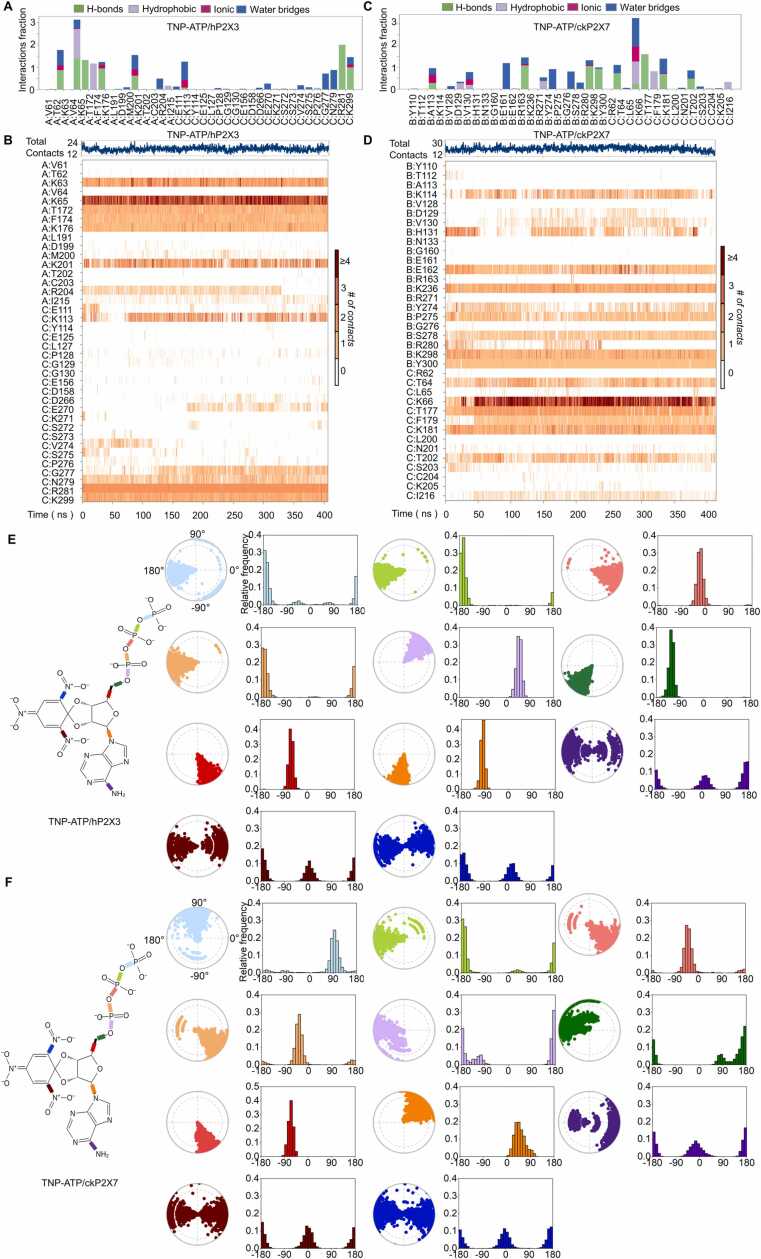
**Conventional molecular dynamics simulations (CMD) of hP2X3/TNP-ATP (PDB ID: 5SVQ) and ckP2X7/TNP-ATP (PDB ID: 5XW6) complexes. (A)** 0.4-μs CMD simulations of hP2X3/TNP-ATP complex showing interactions between key residues and TNP- ATP. The interactions are classified into four types: hydrogen bonds (green), hydrophobic (light purple), ionic (pink) and water bridges (blue). The histogram of the stacking is normalized over the course of the trajectory. **(B)** CMD simulations showing the total interactions of hP2X3 and TNP-ATP. The darker orange color indicates a higher number of residues interacting with the ligand, as some amino acids have multiple specific contracts with the ligand. **(C)** 0.4-μs CMD simulations of the ckP2X7/TNP-ATP complex showing amino acid and TNP-ATP interactions. **(D)** CMD simulations showing the full interaction of ckP2X7 with TNP-ATP. **(E, F)** Conformational evolution of each rotatable bond in the ligand during the simulation trajectory of hP2X3/TNP-ATP (E) and ckP2X7/TNP-ATP (F). Two-dimensional schematics of TNP-ATP are shown as color-coded rotatable bonds. The radial plots represent the conformation of the torsion bodies. The center of the radial plot represents the beginning of the simulation, with the temporal evolution plotted in the radial direction outwardly. The histogram summarizes the data of the corresponding radial plot, which represents the probability density of the torsion.

To further investigate whether these two conformations are only a structural snapshot (transient state) or represent relatively stable conformations, we performed 0.7–1 µs CMD simulations. The conformations of both structures remained relatively stable at this time scale of simulations. For the hP2X3/TNP-ATP structure (PDB ID:5SVQ), TNP-ATP interacted with ^h3^K113, ^h3^E270, ^h3^G277, ^h3^N279, ^h3^R281, ^h3^K299, ^h3^K63, ^h3^K65, ^h3^T172, ^h3^F174, ^h3^K176, ^h3^K201, ^h3^R204 and ^h3^I215 of hP2X3 (Fig. 2A, B); in contrast, for ckP2X7 (PDB ID: 5XW6), TNP-ATP interacted with ^ck7^K114, ^ck7^D129, ^ck7^V130, ^ck7^H131, ^ck7^E162, ^ck7^K236, ^ck7^Y274, ^ck7^P275, ^ck7^S278, ^ck7^R280, ^ck7^K298, ^ck7^Y300, ^ck7^T64, ^ck7^K66, ^ck7^T177, ^ck7^F179, ^ck7^K181, ^ck7^T202 and ^ck7^I216 (Fig. 2C, D). Moreover, the statistics of the evolution of the various bond angles of TNP-ATP throughout CMD simulations indicate that they are mostly constrained in hP2X3 (Fig. 2E) and in ckP2X7 (Fig. 2F) and could only vary within a specific range of values, except for very few groups such as nitro substituents (see below).

These results suggest that the two conformations determined by structural biology have some degree of stability, which could represent either stable or semi-stable intermediate states. At least during CMD simulations at a time scale of μs, these two conformations did not undergo significant conformational changes or state transitions. However, since some of the structures were obtained after purification of the proteins by soaking with the ligand (commonly used to obtain structures of complexes [28], [29], [30]), such as hP2X3 [28], it cannot be ruled out that the obtained conformation only existed under conditions of the structural determination.

### 3.2 The pocket formed by the LF and LB domains is not essential for TNP-ATP recognition of hP2X3

**Fig. 3:**
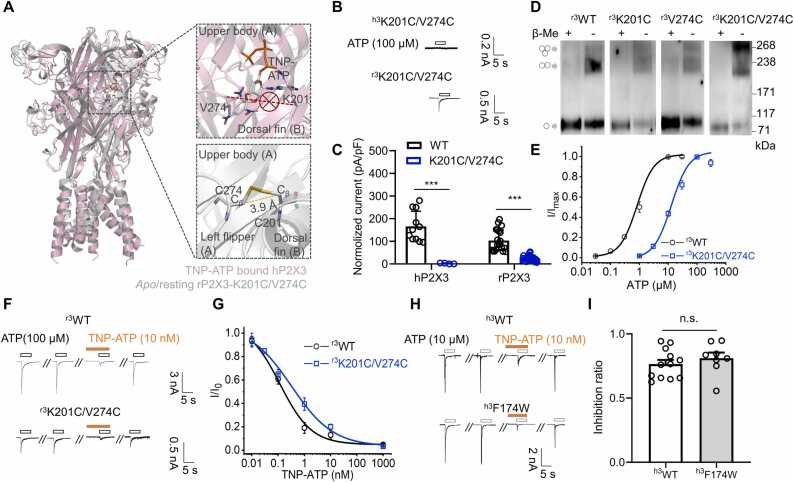
**Engineered disulfide crosslinking together with mutagenesis to validate the interaction between the TNP moiety and hP2X3 suggested by the hP2X3/TNP-ATP crystal structure.** (**A**) Schematic representation of engineered disulfide crosslinking mutant, rP2X3 K201C/V274C (^r3^K201C/V274C). TNP-ATP bound hP2X3-WT (^h3^WT), pink; *apo*/resting ^r3^K201C/V274C, gray. (**B, C**) Representative traces (B) and pooled data (C) recorded from cells transfected with ^h3^K201C/V274C and ^r3^K201C/V274C mutants. ^h3^WT, 166 ± 20 pA/pF (n = 11); ^h3^K201C/V274C, 2.09 ± 1.21 pA/pF (n = 4); ^r3^WT, 103 ± 10 pA/pF (n = 21); ^r3^K201C/V274C, 24.4 ± 2.2 pA/pF (n = 28); ^***^ p < 0.001, unpaired t-test. (D) Homo-oligomers of hP2X3 with double mutations K201C and V274C showed predominantly as trimers in non-reducing Western blots. Cells transfected with ^r3^WT and the cysteine substitution mutants as indicated were lysed in buffers with (+) and without (-) β-Mercaptoethanol (β-Me). Positions corresponding to the sizes of monomeric, dimeric, and trimeric P2X3 subunits are marked with arrows, respectively. (E) ATP dose response curves of ^r3^WT and ^r3^K201C/V274C. EC_50_ of ATP: ^r3^WT, 0.832 ± 0.143 μM, n = 3; ^r3^K201C/V274C, 12.9 ± 2.7 μM, n = 3. (F, G) TNP-ATP inhibitory effects (F) and dose response curves (G) of ^r3^WT (IC_50_ = 0.117 ± 0.092 μM, n = 3) and ^r3^K201C/V274C mutant (0.362 ± 0.071 μM, n = 3). (H, I) Representative currents (H) and pooled data (I) showing TNP-ATP inhibition of ^h3^WT (inhibition ratio = 0.764 ± 0.033, n = 12) and ^h3^F174W (0.810 ± 0.044, n = 8), no significance (n.s.), p > 0.05, unpaired t-test. Each solid line is a fit of the Hill 1 equation. All summary data are expressed as mean ± s.e.m.

To validate the recognition mechanism of TNP-ATP in the hP2X3/TNP-ATP complex, we introduced a pair of cysteine residues (^h3^K201C/V274C) between the LF and DF domains of the P2X3 receptor, which can form inter-subunit disulfide bonds and is theoretically able to block the insertion of the TNP group into the gap between the LF and DF domains (Fig. 3A), thereby reducing the inhibitory effect of TNP-ATP. Since the expression efficiency and ATP-evoked current of the ^h3^K201C/V274C double mutant was markedly decreased, we chose rat P2X3 (rP2X3) as a replacement (Fig. 3B, C), whose orthosteric site and sequences of the LF and DF domain are almost identical to hP2X3 (Fig. S1).

Western blots obtained from non-reducing SDS-PAGE gels showed that ^r3^K201C/V274C exhibited more trimers with a molecular weight of ∼268 kDa as compared to ^r3^wild type (^r3^WT) and single cysteine mutants, which disassembled into monomers of ∼90 kDa after breaking the disulfide bond with β-mercaptoethanol (β-Me) (Fig. 3D). This indicated that disulfide bonds could be formed between subunits. Compared with ^r3^WT, the affinity of ^r3^K201C/V274C to ATP was reduced by about 15-fold (EC_50_ for ATP: ^r3^WT, 0.832 ± 0.140 μM; ^r3^K201C/V274C, 12.9 ± 2.7 μM_;_ Hill 1 function fit; Fig. 3E), which is consistent with the conclusion that S275 of the LF domain is involved in the recognition of ATP [28], [48]. Because TNP-ATP is a competitive inhibitor, to avoid false positives/false negatives associated with agonist nonsaturation, we chose a saturating concentration of 100 μM ATP to examine the inhibition of ^r3^K201C/V274C by TNP-ATP, and compared it with ^r3^WT stimulated with a saturating concentration of 10 μM ATP. Even with a 10-fold increase in ATP concentration for the drug to compete, the apparent inhibitory efficiency of TNP-ATP in ^r3^K201C/V274C was only mildly decreased compared to ^r3^WT (IC_50_: ^r3^WT, 0.117 ± 0.092 nM; ^r3^K201C/V274C, 0.362 ± 0.071 nM; Hill 1 function fit; Fig. 3F, G).

In addition, we evaluated the contribution of ^h3^F714, the only residue showing interaction with the TNP group in the hP2X3/TNP-ATP structure. Since it has been reported that the F 174 A mutant of hP2X3 does not alter the inhibition by TNP-ATP [49], we designed a tryptophan mutant (^h3^F174W) to create a bulkier side chain. However, the inhibition by TNP-ATP remained unchanged (inhibition ratio = 0.810 ± 0.044 for ^h3^F174W, p > 0.05 *vs* ^h3^WT 0.764 ± 0.033, n = 8–12, unpaired t-test; Fig. 3H, I), indicating that ^h3^F174 indeed has no significant contribution to the inhibition by TNP-ATP. These results suggest that the site formed by the LF and LB domains might not contribute to TNP-ATP recognition by hP2X3.

### 3.3 The critical region of hP2X3 that houses the TNP moiety of TNP-ATP is located in the lumen between the head and DF domains

**Fig. 4:**
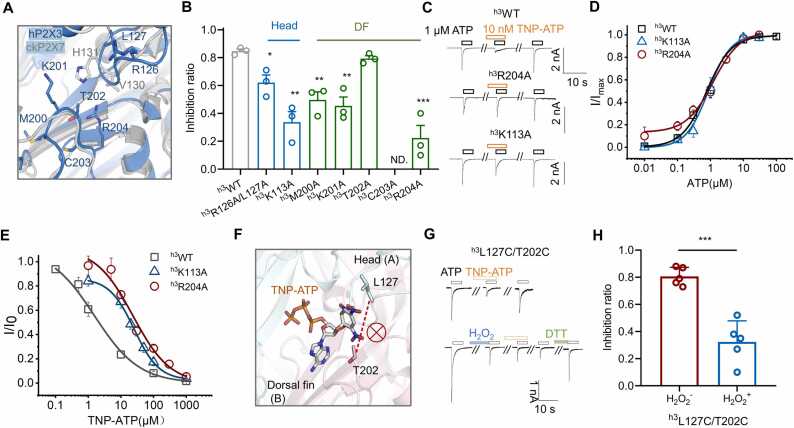
**The lumen between the head and DF domains is crucial for the recognition of TNP- ATP by hP2X3.** (**A**) Superimposition of the head and DF domains of hP2X3 (cyan) and ckP2X7 (gray). The mutated residues are indicated with sticks for emphasis. (**B, C**) Altered inhibition of TNP-ATP in some mutants of the head and DF domains of the hP2X3 receptor (1 μM ATP and 10 nM TNP-ATP). Inhibition ratios: ^h3^WT, 0.847 ± 0.015, n = 3; ^h3^R126A/L127A, 0.623 ± 0.055, n = 3; ^h3^K113A, 0.337 ± 0.075, n = 3; ^h3^M200A (0.497 ± 0.058, n = 3; ^h3^K201A, 0.453 ± 0.064, n = 3; ^h3^T202A, 0.793 ± 0.020, n = 3; ^h3^R204A, 0.223 ± 0.091, n = 3; *p < 0.05, ^**^p < 0.01, ^***^p < 0.001 *vs*. ^h3^WT, one-way ANOVA followed by Dunnett’s multiple comparisons, F (2, 6) = 21.95. (**D, E**) ATP dose response curves (**D)** and TNP-ATP dose inhibition curves (E) of ^h3^WT, as well as ^h3^K113A and ^h3^R204A mutants. ATP EC_50_^: h3^WT, 0.767 ± 0.099 μM, n = 3; ^h3^K113A, 0.872 ± 0.232 μM, n = 3; ^h3^R204A, 1.22 ± 0.22 μM^,^ TNP-ATP IC _50_: ^h3^WT, 1.33 ± 0.58 nM, n = 3; ^h3^K113A, 25.4 ± 1.7 nM, n = 3; ^h3^R204A, 23.3 ± 10.0 nM, n = 3; Hill 1 function fitting. (**F-H**) Schematic (F), representative currents (G), and summarized inhibition ratio (H) showing altered TNP-ATP inhibition of ^h3^L127C/T202C before and after the H_2_O_2_ treatment to induce a disulfide bond formation. Inhibition ratios: before H_2_O_2_ treatment (H_2_O_2_^-^), 0.806 ± 0.032, n = 5; after H_2_O_2_ treatment (H_2_O_2_^+^), 0.324 ± 0.069, n = 5; ^***^ p < 0.001, unpaired t-test .

In contrast, when we screened residues in the head and DF domains by alanine scanning (Fig. 4A, B), we found multiple amino acids in these domains to be important for TNP-ATP inhibition of hP2X3. Mutations in the head domain, ^h3^R126A/L127A and ^h3^K113A, had inhibition ratios 0.623 ± 0.051 and 0.337 ± 0.083, respectively (p < 0.05 and p < 0.01 *vs*. ^h3^WT 0.847 ± 0.015, one-way ANOVA followed by Dunnett's multiple comparisons, n = 3, F (2, 6) = 21.95; Fig. 4B) and that in the DF domain, ^h3^M200A, ^h3^K201A, and ^h3^R204A had inhibition ratios 0.497 ± 0.058, 0.453 ± 0.062, and 0.223 ± 0.091, respectively (p < 0.01, p < 0.01, and 0.001 *vs*. ^h3^WT 0.847 ± 0.015, one-way ANOVA followed by Dunnett's multiple comparisons, n = 3, F (4, 10) = 20.24, Fig. 4B) all significantly attenuated the inhibitory effect of TNP-ATP on hP2X3, especially with the substitutions of ^h3^K113 in the head domain and ^h3^R204 in the DF domain.

Since the ^h3^K113A and ^h3^R204A mutants had little changes in the apparent affinity to ATP (EC_50_: ^h3^WT, 0.767 ± 0.099 μM; ^h3^K113A, 0.872 ± 0.23 μM; ^h3^R204A, 1.22 ± 0.22 μM; Hill 1 function fit; Fig. 4C, D), we chose a saturating concentration of 10 μM ATP for the subsequent TNP-ATP inhibition test. The inhibition efficiency of TNP-ATP was reduced by 19-fold for ^h3^K113A and 18-fold for ^h3^R204A (IC50^:^^h3^WT, 1.33 ± 0.58 nM; ^h3^K113A, 25.4 ± 1.7 nM; ^h3^R204A, 23.3 ± 10.0 nM; Hill 1 function fit; Fig. 4C, E). These changes are more pronounced than that produced by mutations in the LF and LB domains made according to the structure of the hP2X3/TNP-ATP complex (Fig. 3F-I).

To further verify the role of the head and DF domains of hP2X3 in TNP-ATP recognition, we introduced a pair of disulfide bonds, ^h3^L127C/T202C (Fig. 4F), in these two regions. If these two regions are involved in TNP-ATP recognition, the disulfide bond would strongly weaken the inhibitory effect of TNP-ATP. Indeed, the ^h3^L127C/T202C mutant displayed a significantly reduced inhibition by TNP-ATP (inhibition ratio = 0.324 ± 0.069 for H_2_O_2_^+^ (disulfide formed) *vs*. H_2_O_2_^-^ 0.806 ± 0.032, n = 5, p < 0.001, unpaired t-test; Fig. 4G, H^).^ Thus, the head and DF domains of hP2X3, rather than the LB and LF regions shown by the structure of the hP2X3/TNP-ATP complex, actually play an important role in TNP-ATP recognition.

### 3.4 Ehanced conformational sampling by metadynamics (MetaD) simulations demonstrates low relative free energy with the TNP moiety of TNP-ATP bound at the head and DF domains of hP2X3

**Fig. 5:**
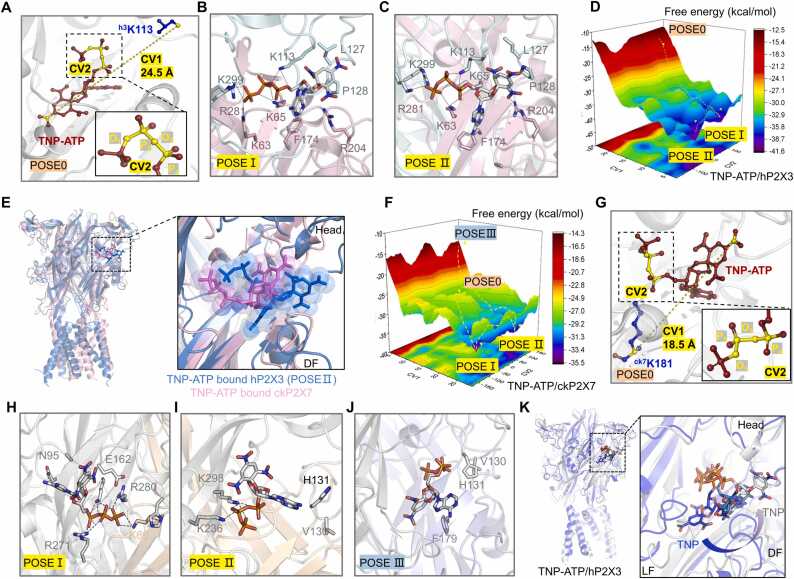
**Enhanced sampling by metadynamics (MetaD) shows relatively low binding free energy when TNP moiety is accommodated by the head and DF domains of the hP2X3 and ckP2X7 receptors. (A)** Definition of two collective variables (CVs), CV1 (yellow) and CV2 (yellow), in MetaD simulations of hP2X3/TNP-ATP (PDB ID: 5SVQ). The values of distance (CV1) and dihedral angle (CV2) are derived from the structure of hP2X3/TNP-ATP. **(B, C)** Two binding poses of TNP-ATP with lower relative free energy in the MetaD trajectory of hP2X3/TNP-ATP. **(D)** Three-dimensional (3D) reconstruction of the free energy surface (FES) based on MetaD simulations. POSE 0, POSE I and POSE II correspond to ∆G of + 14.79, + 1.59 and 0 kcal/mol, respectively. **(E)** The superimposition of the sampled conformation of hP2X3 (POSE II, lowest free energy, blue) and the structure of the ckP2X7/TNP-ATP complex (PDB ID: 5XW6, pink), showing the similarity of the TNP-molecule orientation after MetaD simulations of hP2X3 and ckP2X7. **(F)** 3D reconstruction of the FES. ∆G values are + 5.71, 0, + 0.03 and + 14.94 kcal/mol for POSE 0, POSE I, POSE II, and POSE III, respectively. **(G)** Definition of two collective variables (CVs), CV1 (yellow) and CV2 (yellow), during MetaD simulations of the ckP2X7/TNP-ATP complex (PDB ID: 5XW6). The values of distance (CV1) and dihedral angle (CV2) are derived from the structure of ckP2X7/TNP-ATP. **(H, I)** Two binding poses with lower relative free energy in the MetaD trajectory of ckP2X7/TNP-ATP. **(J)** POSE III derived from the MetaD trajectory of ckP2X7/TNP-ATP showing a similar. TNP-molecule orientation to hP2X3/TNP-ATP, but with higher free energy. (**K**) TNP-group moving from the ATP-binding pocket to the interface between the head and DF domains, as revealed by superimposing the initial (dark blue) and final (gray) poses after 120 ns of MetaD simulations.

The above results implied a different TNP-ATP recognition mode than that revealed by the structure of the hP2X3/TNP-ATP complex. Since the CMD simulations on µs time scale (Fig. 2) could not detect a large conformational change or allow transition to another state, we employed MetaD simulations for enhanced conformational sampling [50]. This method allowed not only the transition from the TNP moiety being accommodated by the LF and LB regions to that by the head and DF domains, but also an evaluation of the relative binding free energies between them. Since the distance will alter significantly if TNP-ATP flips, we chose the distance between the O atom on the ^h3^K113 backbone of the hP2X3 and the N atom on the 4' position nitroxide of TNP as the collective variables 1 (CV1)；the dihedral angle consisting of four consecutive atoms on the backbone of the triphosphate group as CV2 (bright yellow, Fig. 5A) for the accelerated sampling of MetaD simulations. Combinatorial alterations to CV1 and CV2 can affect the relative free energies of the two TNP-group conformations further into the ATP-binding pocket and at the head-DF domain interface.

Based on the 120 ns MeatD simulations and the three-dimensional (3D) reconstruction of the free energy surface, the initial conformation of TNP-ATP (POSE 0) corresponded to a ∆G of + 14.79 kcal/mol, and the two conformations with the lowest free energy, POSE I and POSE II corresponded to ∆G of + 1.59 kcal/mol and 0 kcal/mol, respectively (Fig. 5D). In both conformations with the lowest energy, the TNP moiety was flipped and inserted between the head and DF domains, which is similar to the pattern shown in the ckP2X7/TNP-ATP complex, Fig. 5B, C). We superimposed the lowest energy POSE II conformation with the ckP2X7/TNP-ATP complex (PDB ID: 5XW6) and found that the binding modes of TNP-ATP in both were approximately the same, differing only slightly in the depth of the insertion of the TNP moiety to the gap between the head and DF domains (Fig. 5E).

More importantly, the amino acid residues that interact with the TNP moiety of TNP-ATP in these two conformations with the lowest relative free energy, *i.e*., ^h3^K113 and ^h3^L127 in the head domain and ^h3^R204 in the DF domain, are the same as those we verified above to be important for the TNP-ATP action by analyzing mutants (Fig. 4B, E). This implies that the structurally determined hP2X3/TNP-ATP interaction mechanism (Fig. 1E) likely represents an intermediate state during the interaction of TNP-ATP with hP2X3, rather than a final natural state.

### 3.5 Enhanced conformational sampling by MetaD simulations reveals low relative free energy of the ckP2X7/TNP-ATP complex with the TNP moiety interacting with the head and DF domains

For further inverse validation, we performed MetaD-enhanced sampling on ckP2X7, choosing the distance between the N atom on the nitro group at the 4' position of TNP and the C atom of the ^ck7^K181 backbone in the LF domain as the distance variable CV1, and the four consecutive atoms of the triphosphate group backbone as the dihedral angle variable CV2 (bright yellow, Fig. 5G). And the resulted showed that the ∆G of the initial conformation (POSE 0) was + 5.71 kcal/mol, and that of the two conformations with the free energy minimums were 0 kcal/mol for POSE I and + 0.03 kcal/mol for POSE II (Fig. 5 F). In both conformations, the TNP group of TNP-ATP remains position between the head and DF domains (Fig. 5 H, I). However, the ∆G of POSE III, which resembled the recognition mode of the hP2X3/TNP-ATP complex (*i.e*., TNP interacts with the LB and LF domains, PDB ID: 5SVQ), is + 14.94 kcal/mol, much higher than that of POSE 0, POSE I and POSE II (Fig. 5F, J). By superimposing the initial (dark blue) and final (gray) poses after 120 ns of MetaD simulations, we were able to observe that the TNP-group had moved from the ATP-binding pocket to the interface between the head and DF domains (Fig. 5K). Thus, the enhanced conformational sampling of hP2X3/TNP-ATP and ckP2X7/TNP-ATP by MetaD simulations suggests the recognition of TNP-ATP by both P2X receptors to be more in line with the mode revealed by the ckP2X7/TNP-ATP structure, *i.e*., the interaction of the TNP moiety with the head and DF domains of the P2X receptor.

### 3.6 Residues ^ck7^V130 and ^ck7^H131 in the head domain and mild outward expansion of the TM region contribute to TNP-ATP recognition by ckP2X7

**Fig. 6:**
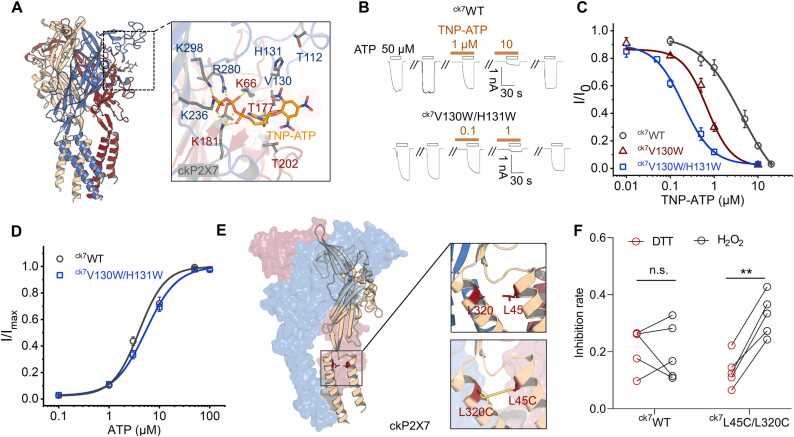
**Residues**^**ck7**^**V130 and**^**ck7**^**H131 in the head domain of ckP2X7 and a slight outward expansion of the transmembrane (TM) region contribute to the recognition of TNP-ATP by ckP2X7.** (**A**) TNP-ATP bound ckP2X7 structure (PDB ID: 5XW6). Residues contributing to TNP-ATP recognition are indicated by sticks for emphasis. (**B, C**) TNP-ATP inhibition (B) and dose response curves (C) for ^ck7^WT, ^ck7^V130W and ^ck7^V130W/H131W. IC_50_: ^ck7^WT, 4.28 ± 0.47 μM, n = 3; ^ck7^V130W, 0.686 ± 0.142 μM, n = 3; ^ck7^V130W/H131W, 0.199 ± 0.036 μM, n = 3. Each solid line is a fit of the Hill 1 equation. (**D**) ATP dose-response curves for ^ck7^WT and ^ck7^V130W/H131W, ^EC^50^: ck7^WT, 3.96 ± 0.78 μM, n = 3; ^ck7^V130W/H131W, 5.36 ± 0.49 μM, n = 3. Each solid line is a fit of the Hill 1 equation. (**E, F**) Schematic showing designed disulfide crosslinking mutants, ^ck7^L45C/L320C (E), and altered TNP-ATP inhibition in this mutant (1 μM TNP-ATP and 50 μM ATP) (F). DTT *vs.* H_2_O_2_^, ck7^WT: 0.234 ± 0.029 *vs.* 0.244 ± 0.067, n = 5, no significance (n.s.), p > 0.05; ^ck7^L45C/L320C: 0.153 ± 0.035 *vs.* 0.384 ± 0*.*027, n = 5, ^**^ p < 0.01, paired t-test. The response of a single cell in paired circumstances is shown by each line.

To validate the information obtained from ckP2X7 interaction with TNP-ATP in MetaD- enhanced conformational sampling, as well as the structure of the ckP2X7/TNP-ATP complex (PDB ID：5XW6), we analyzed mutants with amino acid substitutions in the head and DF domains of ckP2X7. A ^ck7^V130W/H131W double tryptophan mutant was made to restrict the rotation of the TNP moiety, which increased the efficiency of TNP-ATP inhibition of ATP-evoked current by more than 20-fold compared to ^ck7^WT (IC_^50^_^: ck7^WT, 4.28 ± 0.47 μM; ck7V130W/H131W, 0.199 ± 0.036 μM^;^ Hill 1 function fit; Fig. 6A - C). Its apparent affinity to ATP was comparable to ^ck7^WT (EC_50_: ^ck7^WT, 3.96 ± 0.78 μM; ^ck7^V130W/H131W, 5.36 ± 0.49 μM; Hill 1 function fit; Fig. 6D). This indicates that ^ck7^V130 and ^ck7^H131 in the head domain are critical for the recognition of TNP-ATP. The ckP2X7/TNP-ATP and hP2X3/TNP-ATP models differ in the TM region in addition to the TNP-ATP recognition site (Fig. 1F, G): in hP2X3, the TNP-ATP-bound state is similar to the closed state, while in ckP2X7, the TM region of the TNP-ATP-bound state is outwardly expanded, implying that alterations in this region may partially contribute to the binding free energy (including the entropic and enthalpy changes from additional interactions, which are, of course, partially offset by the increased protein strain energy). We reasoned that if the TM region is restricted, it would also affect the inhibitory effect of TNP-ATP on ckP2X7. To test this, we introduced an additional pair of disulfide bond mutants, ^ck7^L45C/L320C (Fig. 6E), in the TM region of ckP2X7. After forming the disulfide bond with the treatment of H_2_O_2_, the inhibition ratio of TNP-ATP was 0.153 ± 0.035, significantly lower than after the disulfide bond was broken with a subsequent treatment of DTT, which had an inhibition ratio of 0.384 ± 0.027 (p < 0.01, paired t-test, n = 3; Fig. 6F). As a control, these treatments did not affect the TNP-ATP inhibition effect on ^ck7^WT (0.234 ± 0.029 *vs*. 0.244 ± 0.067, DTT treatment *vs*. H_2_O_2_ treatment, respectively, p > 0.05, paired t-test, n = 3, Fig. 6F), indicating that the TM region of ckP2X7/TNP-ATP indeed contributes significantly to TNP-ATP inhibition.

### 3.7 Further refinement and confirmation of the interactions revealed by the structure of the ckP2X7/TNP-ATP complex

Next, we explored the contributions of amino acids that form H-bonds with the TNP group as revealed by the ckP2X7/TNP-ATP complex. In the structure, the TNP moiety of TNP-ATP forms H-bonds with ^ck7^T112 (head domain) and ^ck7^T202 (DF domain) (Fig. 1D), which may play a key role in its recognition. To verify this, we first introduced tryptophan at residues ^ck7^T112 and ^ck7^A113 in the head domain to explore the effects of increasing the side chain size on TNP-ATP inhibition.

**Fig. 7:**
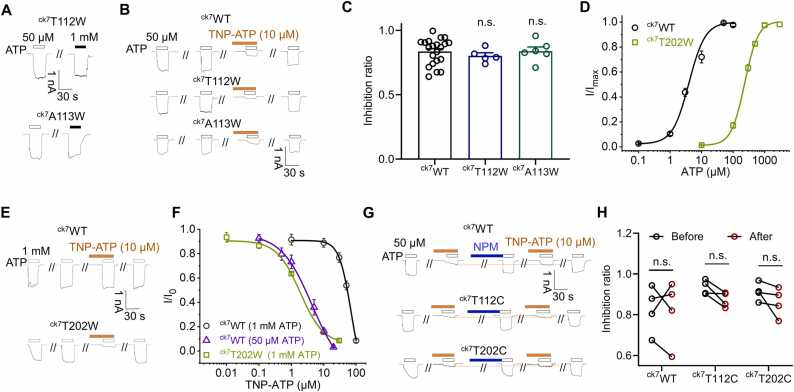
**Mutations of key residues in the structure of the ckP2X7/TNP-ATP complex have no effect on the inhibition of TNP-ATP.** (**A**) ATP responses in ^ck7^WT, ^ck7^T112W and ^ck7^A113W. (**B, C)** Representative current traces (B) and pooled data (C) showing the effect of TNP-ATP (10 μM) on ATP (50 μM)-induced activation in cells transfected with ^ck7^WT or mutants. Inhibition ratios: ^ck7^WT, 0.844 ± 0.022, n = 21; ^ck7^T112W, 0.802 ± 0.023, n = 5; ^ck7^A113W, 0.843 ± 0.033, n = 6; no significance (n.s.), p > 0.05; *p < 0.05 compared to ^ck7^WT, one-way ANOVA followed by Dunnett’s multiple comparisons, F (2, 29) = 0.281. (D) ATP dose response curves of ^ck^7WT (EC_50_ = 3.96 ^±^ 0.78 μM, n = 3) and ^ck7^T202W (232 ± 13 μM, n = 3). Each solid line is a fit of the Hill 1 equation. (E, F) TNP-ATP inhibition (E) and dose-response curves (F) of ^ck7^WT and ^ck7^T202W. IC_50_ of TNP-ATP: ^ck7^WT, 4.28 ± 0.47 μM, 50 μM ATP, n = 3, and 70.9 ± 7.7 μM, 1 mM ATP, n = 3; ^ck7^T202W, 1.87 ± 0.19 μM, 1 mM ATP, n = 3. Each solid line is a fit of the Hill 1 equation. (G, H) Representative current traces (G) and pooled data (H) showing the effect of NPM (1 mM, 5 min) on TNP-ATP inhibition of ^ck7^WT, ^ck7^T112C and ^ck7^T202C: ^ck7^WT, 0.831 ± 0.058 *vs*. 0.822 ± 0.079, n = 4, p > 0.05; ^ck7^T112C, 0.934 ± 0.022 *vs*. 0.878 ± 0.022, n = 4, p > 0.05; ^ck7^T202C, 0.914 ± 0.019 *vs* 0.867 ± 0.035, n = 4, p > 0.05; before *vs*. after NPM modification, paired t-test. The response of a single cell in paired circumstances is shown by each line.

The current magnitudes of ^ck7^T112W and ^ck7^A113W were comparable at ATP concentrations of 50 μM (saturating for ^ck7^WT) and 1 mM (Fig. 7A), indicating that their affinities to ATP did not change significantly. Therefore, we chose 50 μM ATP for subsequent functional experiments. However, compared with ^ck7^WT, there was no significant change in the inhibitory effect of TNP-ATP on ^ck7^T112W and ^ck7^A113W (inhibition ratios = 0.802 ± 0.023 and 0.843 ± 0.033 for ^ck7^T112W and ^ck7^A113W, respectively, p > 0.05 *vs*. WT 0.844 ± 0.022, n = 5–21, one-way ANOVA followed by Dunnett's multiple comparisons, F (2, 29) = 0.281, Fig. 7B, C).

In contrast, the ^ck7^T202W mutant exhibited approximately 60-fold reduction in the affinity to ATP (EC_50_: ^ck7^WT, ^3.96^ ± 0.78 μM; ^ck7^T202W, 232 ± 13 μM; Hill 1 function fit; Fig. 7D); therefore, we chose to activate the mutant channel with 1 mM ATP while testing the inhibition by TNP-ATP. The results showed that the apparent affinity of TNP-ATP was enhanced about 38-fold in ^ck7^T202W compared with ^ck7^WT (^ck7^WT, 70.9 ± 7.7 μM; ^ck7^T202W, 1.87 ± 0.19 μM; Hill 1 function fit; Fig. 7E, F). However, given that its affinity to ATP was also decreased by about 60-fold (Fig. 7D) and that unlike hP2X3, the ckP2X7 receptor displayed a clear competitive interaction between TNP-ATP and ATP (*i.e*., the inhibitory effect of TNP-ATP became significantly weaker with an increased concentration of ATP; Fig. S2A, B), we suspected that the enhanced TNP-ATP inhibition caused by ^ck7^T202W might result from its reduced ATP affinity rather than enhanced TNP-ATP binding.

To verify the above point, we examined the effect of covalent occupancy of ^ck7^T112C and ^ck7^T202C on the inhibition of TNP-ATP using N-phenylmaleimide (NPM, 1 mM, 5 min incubation), which should cause covalent and irreversible attachment of NPM to the cysteine residue, thereby increasing the spatial hindrance at the affected position. However, the effect of TNP-ATP inhibition before and after NPM modification was not significantly altered for either ^ck7^WT, ^ck7^T112C, or ^ck7^T202C (^ck7^WT, 0.831 ± 0.058 *vs*. 0.822 ± 0.079, p > 0.05; ^ck7^T112C, 0.934 ± 0.022 *vs*. 0.878 ± 0.022, p > 0.05; ^ck7^T202C, 0.914 ± 0.019 *vs*. 0.867 ± 0.035, p > 0.05; n = 4; paired t-test; Fig. 7G, H). Thus, amino acids ^ck7^T112 and ^ck7^T202, which are suggested to interact with TNP-ATP in the structure of the ckP2X7/TNP-ATP complex, are not critical for the inhibitory action of the compound on the channel.

Statistics on the evolution of TNP-ATP at individual rotatable bonds (RB) interacting with ckP2X7 during CMD simulations revealed that the two nitro groups of TNP-ATP facing the head and DF domains approached a 360° rotation (Fig. 2F), implying that this position is not restricted by ckP2X7. The ^ck7^V130W/H131W double tryptophan mutant may limit this fluctuation, resulting in a 20-fold increase in TNP-ATP inhibition efficiency (Fig. 6B, C). In contrast, the ^ck7^V130T/H131T mutant with smaller side chains did not cause a significant change in inhibition caused by 10 μM TNP-ATP (inhibition ratio = 0.847 ± 0.069 for ^ck7^V130T/H131T, *vs*. ^ck7^WT 0.844 ± 0.022, n = 4–21, p > 0.05, unpaired t-test, Fig. S3A, B). On the other hand, the dynamic recognition of TNP group may also be one of the reasons for the non-significant effect of the ^ck7^T112C mutant in the head domain and ^ck7^T202C mutant in the DF domain on TNP-ATP inhibition.

In addition, we tried to explore the role of conserved residues in the upper body domain, such as ^ck7^K181 and ^h3^K176 on TNP-ATP inhibition. These residues were shown to interact with TNP-ATP in both the ckP2X7 and hP2X3 crystal structures (Fig. 2). However, mutant made at neither of these sites was functional (Fig. S3C), occluding further functional analysis.

Thus, the structure of the ckP2X7/TNP-ATP complex yielded relatively precise receptor-ligand interactions, showing that the head, DF and TM regions played roles in the inhibition of TNP-ATP. However, the key recognition residues may not be ^ck7^T112 and ^ck7^T202 as shown in the ckP2X7/TNP-ATP complex structure. Functional studies suggest that ^ck7^V130 and ^ck7^H131, located in the head domain, are probably more important.

### 3.8 The lumen between the head and DF domains is equally important for TNP-ATP recognition of other P2X subtypes

**Fig. 8:**
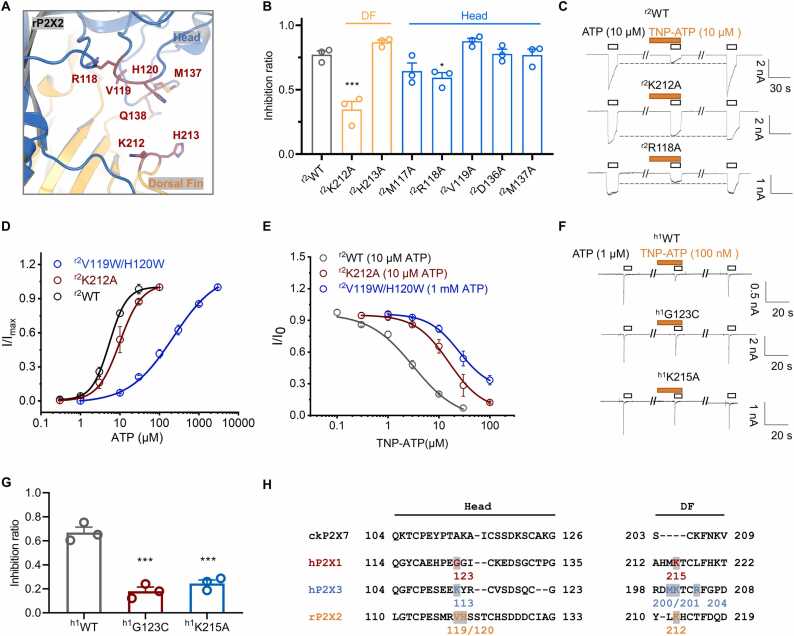
**The lumen between the head and DF domains is also important for TNP-ATP recognition by rat P2X2 (rP2X2) and human P2X1 (hP2X1).** (**A**) Key residues in the head and DF domains of rP2X2. (**B, C)** Pooled data (B) and representative current traces (C) showing the effect of TNP-ATP (10 μM) on ATP (10 μM)-induced currents in ^r2^WT and its mutants. Inhibition ratios: ^r2^WT, 0.771 ± 0.030, n = 3; ^r2^K212A, 0.347 ± 0.064, n = 3; ^r2^H213A, 0.868 ± 0.019, n = 3; ^r2^M117A, 0.644 ± 0.063, n = 3; ^r2^R118A, 0.593 ± 0.039, n = 3; ^r2^V119A, 0.876 ± 0.023, n = 3; ^r2^D136A, 0.778 ± 0.036, n = 3; ^r2^M137A, 0.769 ± 0.046, n = 3; *p < 0.05, ^***^p < 0.001, compared to ^r2^WT, one-way ANOVA followed by Dunnett’s multiple comparisons, F (7, 16) = 16.73. (D, E) ATP dose response curves (D) and TNP-ATP inhibition ^dose-response curve (E) of r2^WT^, r2^K212A and ^r2^V119W/H120W. EC_50_ for ATP: ^r2^WT, 5.39 ± 0.11 μM, n = 3; ^r2^K212A, 9.83 ± 0.34 μM, n = 3; ^r2^V^119W/H120W, 221 ± 36 μM, n = 3; IC^50 ^for TNP-ATP: r2^WT, 3.14 ± 0.95 μM, n = 3; ^r2^K212A,15.8 ± 2.0 μM, n = 3; ^r2^V119W/H120W, 24.9 ± 5.0 μM, n = 3; Hill 1 function fitting. (F, G) Representative currents (F) and pooled data (G) of TNP-ATP inhibition on ^h1^WT and its mutants, ^h1^G123C and ^h1^K215A. Inhibition ratios: ^h1^WT, 0.670 ± 0.044, n = 3; ^h1^G123C, 0.180 ± 0.034, n = 3; ^h1^K215A, 0.245 ± 0.030, n = 3; ^***^p < 0.001 compared to ^h1^WT, one-way ANOVA followed by Dunnett’s multiple comparisons, F (2, 6) = 53.46. (H) Sequence alignment of the head and DF domains of ckP2X7, hP2X1, hP2X3 and rP2X2. Residues contributing to TNP-ATP recognition are highlighted.

Although a similar mechanism exists for TNP-ATP action on hP2X3 and ckP2X7, whether it is also applicable to the recognition of TNP-ATP by other P2X subtypes needed to be further examined. We have verified this being a common mechanism for rat P2X2 (rP2X2, Fig. 8A-E) and human P2X1 (hP2X1, Fig. 8F-G).

In rP2X2, we screened the residues in the head and DF domains and found that mutants ^r2^K212A in the DF domain and ^r2^R118 A in the head domain had reduced inhibition by TNP-ATP (inhibition ratio of ^r2^WT = 0.771 ± 0.030, n = 3; ^r2^K212A = 0.347 ± 0.064, n = 3, p < 0.001; ^r2^R118A = 0.593 ± 0.039, n = 3, p < 0.05; unpaired t-test; Fig. 8A-C). Taking ^r2^K212A as an example, there was no significant change in sensitivity to ATP (EC_50_: ^r2^WT, 5.39 ± 0.11 μM; ^r2^K212A, 9.83 ± 0.34 μM; Hill 1 function fit; Fig. 8D); however, the potency of inhibition by TNP-ATP was reduced by ∼5 fold compared with ^r2^WT (IC_50_: ^r2^WT, 3.14 ± 0.95 μM; ^r2^K212A, 15.8 ± 2.0 μM; Hill 1 function fit; Fig. 8E). In addition, we found that the double mutant ^r2^V119W/H120W reduced sensitivity to TNP-ATP inhibition by ∼8 fold (IC_50_ = ^r2^WT, 3.14 ± 0.95 μM; ^r2^V119W/H120W, 1 mM ATP activation, IC_50_ = 24.9 ± 5.0 μM; Fig. 8D-E), but this mutant also reduced the affinity of rP2X2 to ATP.

Finally, we demonstrated the function of equivalent residues on hP2X1 (^h1^G123 and ^h1^K215, Fig. 8H) in TNP-ATP recognition. Mutants ^h1^G123C and ^h1^K215A showed significantly reduced inhibitory effect of TNP-ATP (inhibition ratio of ^h1^WT = 0.670 ± 0.044, n = 3; ^h1^G123C = 0.180 ± 0.034, n = 3, p < 0.001; ^h1^K215A = 0.245 ± 0.030, n = 3, p < 0.001; unpaired t-test; Fig. 8F- H). Thus, for different species and different subtypes of P2X receptors, TNP-ATP may interact with them through a common mechanism, *i.e*., blocking the opening of the channel by narrowing the cavity between the head and DF domains of the receptor.

## 4 Discussion

Here, we evaluated two distinct modes of TNP-ATP binding to P2X receptors (hP2X3 and ckP2X7) suggested by recent structural studies and propose an improved mechanism of P2X/TNP-ATP action. Our results suggest that TNP-ATP interacts with P2X receptors in a similar pattern to that revealed by the structure of the ckP2X7/TNP-ATP complex, *i.e*., with TNP-ATP embedded in the P2X receptor adapting a "U" shape, similar to ATP, but not a "Y" shape as revealed by the structure of the hP2X3/TNP-ATP complex. TNP-ATP acts by interacting with amino acids in the head and DF domains, where it blocks the opening of the channel by restricting the movement of these two domains. When bound by TNP-ATP, the overall structure of the P2X receptor resembles semi-activation, but without ion permeation.

In the structure of the hP2X3/TNP-ATP complex, the TNP moiety of TNP-ATP is inserted between the LF and LB domains, which could limit channel opening by interfering with the movement of these two regions (Fig. 1E). However, although the introduction of the ^r3^K201C/V274C disulfide bond mutant indeed limited channel opening (Fig. 3C), the affinity to TNP-ATP was only slightly reduced in this mutant (Fig. 3F, G), suggesting that the effect most likely resulted from a weakened binding of the triphosphate group, which is common in both ATP and TNP-ATP, to rP2X3 (Fig. 3E). On the other hand, mutating F174, the only residue suggested by the resolved structure to interact with the TNP moiety of TNP-ATP, to an amino acid with a bulkier side chain also did not affect the inhibitory effect of TNP-ATP (Fig. 3H, I). These results imply that the resolved structure of the hP2X3/TNP-ATP complex may not represent a final state. This is supported by the results of our MetaD simulations, in which the structure of hP2X3/TNP-ATP flipped to a more stable ckP2X7/TNP-ATP-like conformation (POSE I and POSE II) during accelerated conformational sampling, and the TNP moiety that exerts the inhibitory effect interacted with residues in the head and DF domains (Figs. 4A-H and 5A-D). These results suggest that the resolved hP2X3/TNP-ATP structure may differ from the natural ligand recognition. This discrepancy between structural biology and other experiments might be due to the use of ligand soaking (the ckP2X7/TNP-ATP structure was determined by co-crystallization method) or some other conditions during sample preparation; for example, it is known that high salt concentrations and low temperatures affect the ligand-receptor interaction and stabilize it in some unnatural states. We also found high free energy barriers for the ckP2X7/TNP-ATP complex (POSE III, Fig. 5J) when it adapts a structure similar to the resolved hP2X3/TNP-ATP complex during MetaD-enhanced conformational sampling.

The structure of the ckP2X7/TNP-ATP complex showed that the cavity between the head and DF domains of the P2X receptor is the region where the TNP moiety of TNP-ATP is recognized, but mutations at ^ck7^T112 of the head domain and ^ck7^T202 of the DF domain, which show H-bonds with TNP in the crystal structure of the ckP2X7/TNP-ATP complex, did not affect the recognition of TNP-ATP (Fig. 7A- H). Instead, two other amino acids, ^ck7^V130 and ^ck7^H131, which do not show explicit interactions with the TNP moiety in the crystal structure, were found to have an important role in the recognition of TNP-ATP by ckP2X7 (Fig. 6B-D). This difference could result from the following reasons. First, the crystallization conditions might lead to some deviations such that the MetaD stimulation reveals more stable conformations (POSE I and POSE II; Fig. 5H, I) with ^ck7^V130 and ^ck7^H131 as the more critical residues that coordinate the interaction of TNP-ATP with ckP2X7. Second, as shown by CMD simulations, the TNP moiety of TNP-ATP has an important role in the recognition of ckP2X7, which could create multiple transient states during the ligand-receptor interaction (Fig. 2E, F). This means that the TNP-interacting amino acids in the head and DF domains of ckP2X7 may not be fixed and the resolved structure of the ckP2X7/TNP-ATP complex represents just one of the trapped conformations. Third, it cannot be ruled out that at a resolution of 3.1 Å, it is quite difficult to accurately define the side chain orientation of threonine in the ckP2X7/TNP-ATP structure. Therefore, whether the OG1 (oxygen) atom of either of the threonine side chains is actually oriented toward the TNP moiety of TNP-ATP to form a hydrogen bond is still questionable. Nonetheless, despite the details of the amino acids involved in binding to TNP, the contribution of the TM region to TNP-ATP inhibition, which was predicted by the ckP2X7/TNP-ATP complex structure but not the hP2X3/TNP-ATP structure, was confirmed in our study (Fig. 6E, F), demonstrating the relative soundness of the ckP2X7/TNP-ATP interaction mode.

Finally, we showed that equivalent amino acid residues in the head and DF domains of other subtypes of P2X receptors from other species, like rP2X2 and hP2X1, also play important roles in TNP-ATP recognition (Fig. 8A-H), indicating that the mechanism underlying the action of TNP-ATP in P2X receptors is a shared one, *i.e*., by inserting the TNP moiety in between the head and DF domains of the P2X receptor, blocking the conformational change of these two regions, thus preventing the opening of the channel to produce an inhibitory effect. Interestingly, the amino acids identified in the head and DF domains of hP2X3, ckP2X7, rP2X2 and hP2X1 that are critical for TNP-ATP recognition, *i.e*., ^h3^R126/L127, ^h3^K113, ^h3^M200, ^h3^K201, ^h3^R204, ^ck7^V130, ^ck7^H131, ^r2^R118, ^r2^V119, ^r2^H120, ^r2^K212, ^h1^G123 and ^h1^K215, are not conserved, which may stem from species and subtype differences. Particularly, the P2X7 subtype possesses a fragment between β2,3 with a long loop that is absent in all other subtypes [32]. However, residues contributing to TNP-ATP recognition are more conserved in other subtypes with relatively similar structures (hP2X3, rP2X2, and hP2X1). This implies both conserved and specific mechanisms of action of the nonselective inhibitor TNP-ATP on different subtypes of P2X, which may be exploited for the development of subtype-specific competitive inhibitors.

## 5 Conclusion

Taken together, our study reveals a common mechanism of action of TNP-ATP on P2X receptors, opening new avenues for understanding the gating mechanism of P2X receptors and designing new modulators targeting P2X receptors.

## CRediT authorship contribution statement

**Xiao-Bo Ma:** Investigation, Formal analysis, Writing – original draft, Data curation, Visualization. **Chen-Xi Yue:** Investigation, Formal analysis, Writing – original draft, Data curation, Visualization. **Yan Liu:** Investigation. **Yang Yang:** Investigation. **Jin Wang:** Investigation. **Xiao-Na Yang:** Investigation. **Li-Dong Huang:** Investigation. **Michael X. Zhu:** Writing – review & editing. **Motoyuki Hattori:** Writing – review & editing, Funding acquisition. **Chang-Zhu Li:** Writing – review & editing. **Ye Yu:** Conceptualization, Supervision, Formal analysis, Writing – review & editing, Funding acquisition, Project administration. **Chang-Run Guo:** Methodology, Supervision, Investigation, Formal analysis, Writing – review & editing, Funding acquisition, Project administration.

## Declaration of Competing Interest

The authors declare that they have no competing interests in this paper.

## Appendix A Supplementary material

Supplementary material **Fig. S1. Comparison of the ATP-binding pocket and nearby regions between rP2X3 and hP2X3 receptors.** (**A**) Sequence alignment of rP2X3 (blue) and hP2X3 (orange). The sequences of the lower body, left flipper and upper body domains are identical except for a few residues in the dorsal fin region (red). (**B**) Superimposition of the structure of hP2X3 (orange) and the homology model of rP2X3 (blue). **Fig. S2. Slightly different competitive inhibitory activities of TNP-ATP on hP2X3, ckP2X7 and rP2X2.** (**A, B**) Typical traces (A) and pooled data (B) showing the different inhibition patterns of TNP-ATP on hP2X3, ckP2X7 and rP2X2. Inhibition ratios in just-saturated ATP *vs*. tenfold-saturated ATP: hP2X3, 0.764 ± 0.033 (n = 12) *vs*. 0.896 ± 0.023 (n = 5), *p < 0.05; ckP2X7, 0.844 ± 0.022 (n = 21) *vs*. 0.356 ± 0.031 (n = 7), ^***^p < 0.001; rP2X2, 0.726 ± 0.032 (n = 5) *vs*. 0.186 ± 0.061 (n = 4), ^***^p < 0.001; unpaired t-test. **Fig. S3. Non-polar residues V130 and H131 are essential for TNP-ATP recognition of ckP2X7. (A, B)** Representative current traces (A) and pooled data (B) showing the effect of TNP-ATP (10 μM) on channel activation induced by ATP (50 μM or 1 mM) in ^ck7^WT and ^ck7^V130T/H131T. Inhibition ratios: ^ck7^WT, 0.844 ± 0.022, n = 21; ^ck7^V130T/H131T, 0.847 ± 0.069, n = 4; no significance (n.s.), ^p > 0.05^; unpaired t-test. (**C**) ATP response of ^ck7^K181L and ^h3^K176L mutants.
