# Supplementary material for: A shared mechanism for TNP-ATP recognition by members of the P2X receptor family
Source: Comput Struct Biotechnol J. 2023 Dec 7;23:295–308. doi: 10.1016/j.csbj.2023.12.005 (PMC10762375; doi:10.1016/j.csbj.2023.12.005)
Supplement: Supplementary file 1 — Supplementary material Fig. S1. Comparison of the ATP-binding pocket and nearby regions between rP2X3 and hP2X3 receptors. (A) Sequence alignment of rP2X3 (blue) and hP2X3 (orange). The sequences of the lower body, left flipper and upper body domains are identical except for a few residues in the dorsal fin region (red). (B) Superimposition of the structure of hP2X3 (orange) and the homology model of rP2X3 (blue). Fig. S2. Slightly different competitive inhibitory activities of TNP-ATP on hP2X3, ckP2X7 and rP2X2. (A, B) Typical traces (A) and pooled data (B) showing the different inhibition patterns of TNP-ATP on hP2X3, ckP2X7 and rP2X2. Inhibition ratios in just-saturated ATP vs. tenfold-saturated ATP: hP2X3, 0.764 ± 0.033 (n = 12) vs. 0.896 ± 0.023 (n = 5), *p < 0.05; ckP2X7, 0.844 ± 0.022 (n = 21) vs. 0.356 ± 0.031 (n = 7), ***p < 0.001; rP2X2, 0.726 ± 0.032 (n = 5) vs. 0.186 ± 0.061 (n = 4), ***p < 0.001; unpaired t-test. Fig. S3. Non-polar residues V130 and H131 are essential for TNP-ATP recognition of ckP2X7. (A, B) Representative current traces (A) and pooled data (B) showing the effect of TNP-ATP (10 μM) on channel activation induced by ATP (50 μM or 1 mM) in ck7WT and ck7V130T/H131T. Inhibition ratios: ck7WT, 0.844 ± 0.022, n = 21; ck7V130T/H131T, 0.847 ± 0.069, n = 4; no significance (n.s.), p > 0.05; unpaired t-test. (C) ATP response of ck7K181L and h3K176L mutants. [file mmc1.pdf]

Fig. S1. by Xiao-Bo Ma & Chen-Xi Yue et al.

A

	Lower body	Lower body
rP2X3	62TKVKG66	171FTIFIK176
hP2X3	62TKVKG66	171FTIFIK176

	Left flipper	Dorsal fin
rP2X3	273SVSPG277	200IKRC203
hP2X3	273SVSPG277	200MKTC203

	Upper body
rP2X3	278YNFRFAKYYKMENGSEYRTLLKA300
hP2X3	278YNFRFAKYYKMENGSEYRTLLKA300

B

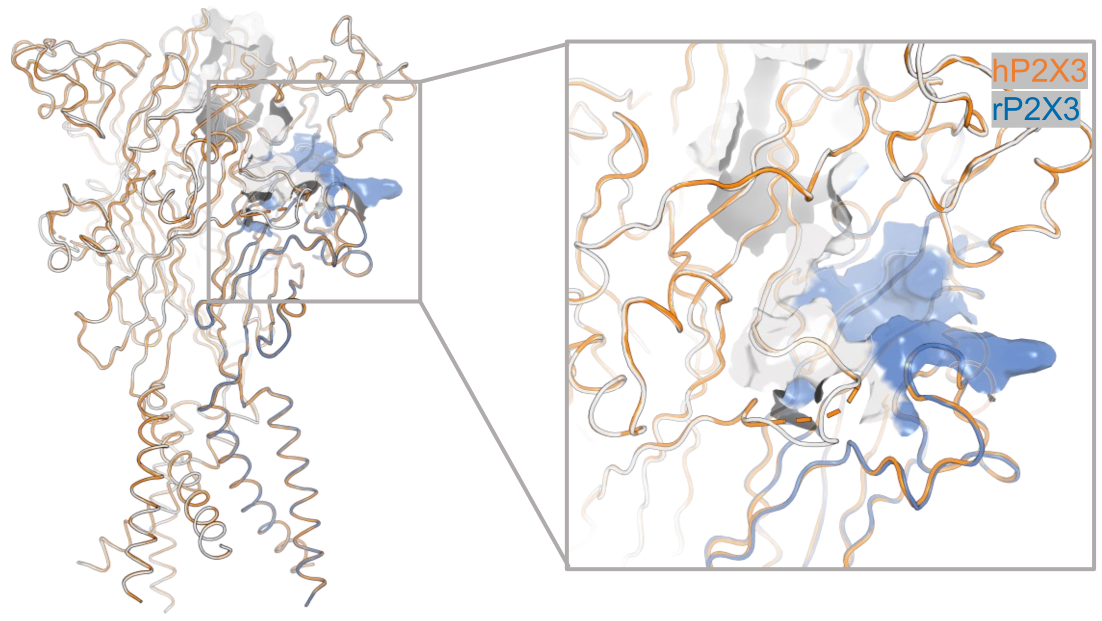

Fig. S2. by Xiao-Bo Ma & Chen-Xi Yue et al.

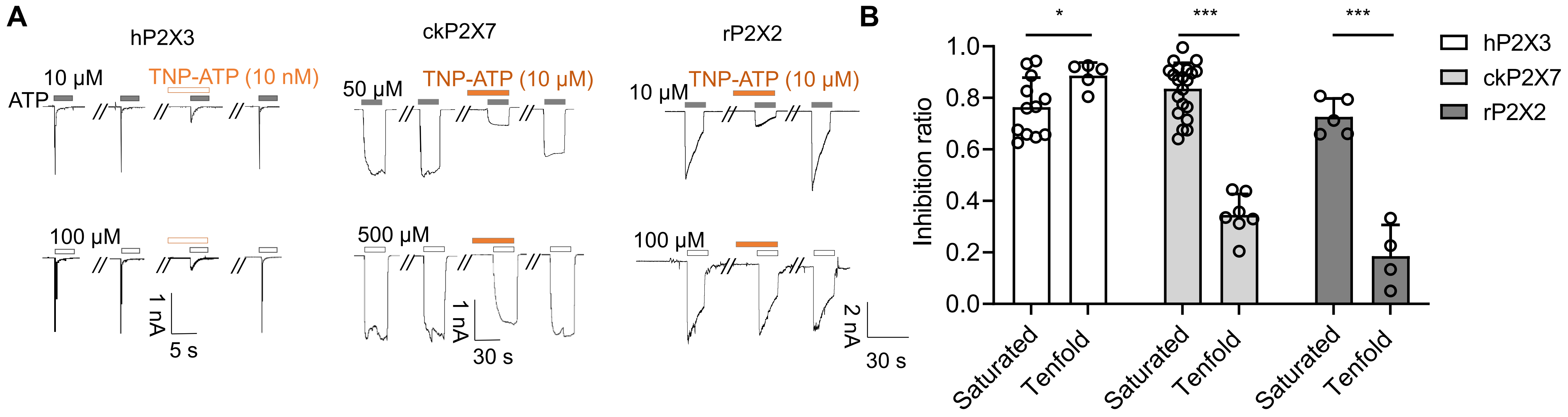

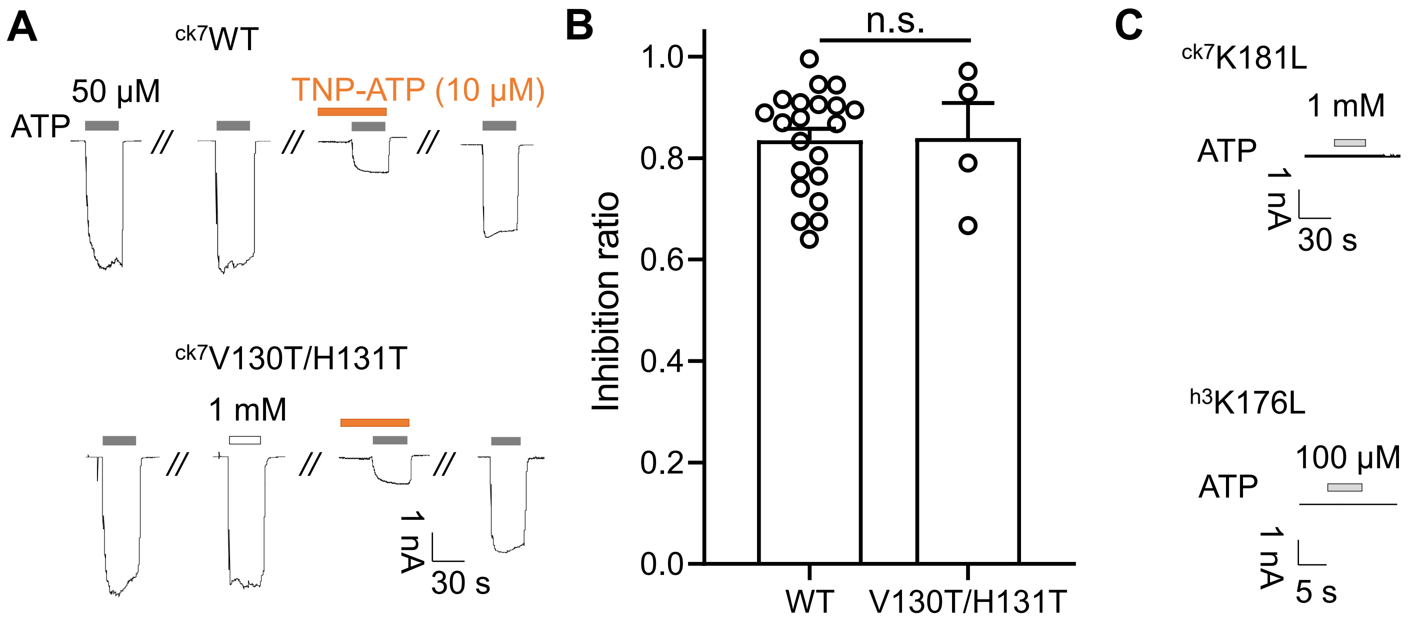
A
ck7WT
50 μM
ATP
TNP-ATP (10 μM)
ck7V130T/H131T
1 mM
1 nA
30 s
B
n.s.
Inhibition ratio
1.0
0.8
0.6
0.4
0.2
0.0
WT
V130T/H131T
C
ck7K181L
1 mM
ATP
1 nA
30 s
h3K176L
100 μM
ATP
1 nA
5 s
